# Delusional Misidentification Syndromes and Violent Offending: A Systematic Review of the Literature

**DOI:** 10.1192/bjo.2025.10107

**Published:** 2025-06-20

**Authors:** Corinne Selin Calkam, Artemis Igoumenou, Vaughan Bell

**Affiliations:** ^1^University College London, London, United Kingdom; ^2^Queen Mary University of London, London, United Kingdom

## Abstract

**Aims:** Delusional misidentification syndromes (DMS) are characterised by a delusional belief of misidentification concerning familiar individuals, places or objects and by the conviction that they have been replaced or transformed. Violent behaviours towards the “impostor” are often observed and can take the form of verbal threats or physical assault. This review explores the specific factors that increase the risk of violence in individuals with DMS.

**Methods:** An initial search was conducted in PsycInfo, MEDLINE, PubMed and PsycArticles in May 2023, followed by a subsequent search in November 2024, to identify publications reporting severe violence (e.g. homicide, attempted murder, assault) in individuals with DMS. 13 papers comprising 16 case reports were included in the review.

**Results:** The majority of patients were male (N=15), aged 29–43 (i.e. early- to mid-adult years) (N=14) at the time of the offence and had a prior diagnosis of a psychiatric disorder (N=13) (i.e. psychotic disorder). In 13 of 16 cases, the DMS was Capgras syndrome. The violent act most commonly perpetrated was homicide (including uxoricide, matricide, patricide, parricide and filicide) (N=21). Victims were mostly acquaintances or strangers (N=16), followed closely by familiar individuals (N=13). In 13 cases, social behaviour of the patients prior to the offence was described as “hostile”, “aggressive”, “solitary” or involving “poor social interactions from a young age”. Only 3 patients were described as “lively” or “social”.

**Conclusion:** The current systematic review identified specific factors such as a prior psychiatric diagnosis (i.e. of psychotic disorders), as well as male gender, early- to mid-adulthood, the type of DMS (i.e. Capgras syndrome) and social behaviour marked by isolation and hostility prior to the offence as potential contributors to severe violence in individuals with DMS. However, the lack of available case reports and empirical studies makes it challenging to understand the psychopathology exhibited and its relationship with violent behaviour. Further research is needed to advance our understanding of the possible factors associated with and the possible causes underlying the severity of violence exhibited in DMS.

